# 한국 간호대학생의 부검에 대한 태도에 영향을 미치는 요인: 횡단적 조사연구

**DOI:** 10.7586/jkbns.24.039

**Published:** 2025-02-21

**Authors:** Seonmi Nho, Hanna Choi

**Affiliations:** 1 Gwangju Metropolitan Police Agency Criminal Division, Gwangju, Korea; 1 광주경찰청 형사과 과학수사계; 2 Department of Nursing Science, Nambu University, Gwangju, Korea; 2 남부대학교 간호학과

**Keywords:** Nursing students, Autopsy, Attitude, 간호대학생, 부검, 태도

## Abstract

**Purpose:**

The purpose of this study was to investigate nursing students’ perceptions of and attitudes toward autopsies and to identify factors influencing their attitudes.

**Methods:**

This study was a descriptive survey, and the participants were 183 nursing students enrolled at Nambu University. Data were collected through an online survey conducted from September 2 to October 4, 2024 and analyzed using SPSS version 28.0, employing descriptive statistics, the t-test, one-way analysis of variance, the Scheffé test, the Pearson correlation coefficient, and multiple regression analysis.

**Results:**

Nursing students’ attitudes toward autopsies showed significant correlations with their awareness of autopsies (β = .29, *p* < .001), awareness of local installation of autopsy facilities (β = .16, *p* = .025), the perceived necessity of forensic medicine (forensic nursing) (β = .16, *p* = .028), and age (β = .12, *p* = .098), explaining a total of 17% of the variance.

**Conclusion:**

These findings underscore the need to develop and implement a systematic forensic education program to improve nursing students’ perceptions of autopsies and facilitate informed decision-making.

## 서론

### 1. 연구의 필요성

현대 복지국가에서 사람들의 죽음은 다양한 원인으로 발생하며, 죽음의 형태와 상황은 개인의 생활방식, 건강상태, 사회적•경제적 요인 등 여러 요소에 따라 다양하게 나타난다[1]. 이러한 다양성은 죽음을 예측하기 어렵게 만들며, 과학적이고 정확한 사망 원인 규명이 필수적이다.

부검은 사망 원인을 규명하는 가장 과학적이고 정확한 방법으로, 사체를 조사하여 병변과 손상을 파악하고 그 원인과 정도를 분석하여 사망과의 인과관계를 밝히는 작업을 의미한다[2,3]. 부검은 대량재난사고, 보험사고, 교통사고, 산업재해 등 다양한 상황에서 국민의 정당한 권리를 보호하고, 범죄로 인한 사망의 경우 범죄의 재발 방지에도 기여한다[4]. 또한 설명되지 않은 질병으로 인한 사망의 경우 부검은 사망의 원인을 규명할 수 있으며, 유전 질환이 원인으로 밝혀질 경우 유족에게 조기 진단과 예방 정보를 제공한다[5]. 이처럼 부검은 개인의 건강관리뿐만 아니라 공중보건 향상과 지역사회의 지속 가능성 있는 발전에도 기여한다[6,7].

최근 국립과학수사연구원의 감정처리 현황에 따르면 부검이 요구되는 사례가 2020년 581,796건, 2021년 651,066건, 2022년 700,856건으로 증가하고 있다[8]. 그러나 우리나라에서는 대한법의학회 변사 가이드라인과 경찰청 훈령의 변사사건 처리 규칙에서 부검의 필요성과 중요성을 명시하고 있음에도 불구하고, 검시대상이 법률적으로 명확히 규정되지 않은 실정이다[9,10]. 더욱이 부검이 유가족들에게 두벌죽음으로 인식되어 고인에 대한 예의에 반하는 행위로 여겨지는 국민 정서도 부검 시행을 어렵게 한다[11,12]. 이로 인해 타살 사망이 자살로 판명되거나, 유전 질환의 진단 기회가 상실되는 등의 문제가 발생할 가능성이 있다[13]. 그러나 유족들의 법적 권리에 대해 관심도 높아지고 한층 지식수준도 높아지면서 부검에 대한 인식 개선과 제도적 변화가 요구되고 있다[14].

간호사는 임상, 지역사회, 학교 등 다양한 환경에서 죽음 및 유가족의 부검 결정에 영향을 주는 상황에 자주 직면할 수 있다. 이 과정에서 간호사는 부검과 관련된 개념을 명확히 이해하고, 유가족에게 신뢰를 주며 불확실성을 줄일 수 있어야 한다[15]. 특히, 간호사는 환자와 많은 시간을 함께하며 환자의 대변인, 교육자, 옹호자, 상담자로서의 역할을 수행하기 때문에, 부검과 관련된 정확한 정보를 전달하고 유족들의 의사결정을 지원할 수 있는 역량이 필요하다.

검시조사관은 이러한 간호사의 역할 중 하나로[16], 간호사들이 법의학 지식을 바탕으로 변사체 및 주변 환경을 조사하여 범죄 관련성을 판단하고, 부검의 필요성을 평가하는 직무를 수행한다[17]. 현재 검시조사관의 약 60%가 간호사로 구성되어 있으며(279명 중 166명)[18], 이는 간호사의 역할이 점차 전문화되고 있음을 보여준다[19].

선행연구에서 간호사[20,21], 의료기관 종사자[20,22], 일반인[23], 의과대학생[24-26], 간호대학생[27]을 대상으로 부검의 인식과 태도를 조사한 바 있었다. 그러나 최근 간호대학생을 대상으로 조사한 부검 관련 연구는 드물며, 2011년과 2014년 사이에 조사한 부검의 필요성과 부검의 거부감 정도, 간호대학생의 부검에 대한 태도와 법의학(법의간호학) 교육의 효과에 그치는 실정이다[13,27]. 간호대학생들이 임상 및 지역사회에서 전문가로 성장하기 위해서는 부검에 대한 올바른 인식과 태도가 형성될 필요가 있다[27,28].

이에 본 연구는 간호대학생을 대상으로 부검에 대한 인식과 태도를 파악하여, 향후 간호교육과정에서 기초교육 강화의 일환으로 부검과 관련한 체계적인 교육과정 개발을 위한 기초 자료를 제공하고자 한다.

### 2. 연구 목적

본 연구의 목적은 간호대학생의 부검에 대한 인식과 태도를 파악하고, 부검에 대한 태도에 미치는 영향을 알아보는 것으로 구체적인 목적은 다음과 같다.

1) 간호대학생의 일반적 특성을 파악한다.

2) 간호대학생의 부검에 대한 인식과 태도 정도를 파악한다.

3) 간호대학생의 일반적 특성에 따른 부검에 대한 인식과 태도의 차이를 파악한다.

4) 간호대학생의 부검에 대한 인식과 태도 간의 상관관계를 파악한다.

5) 간호대학생의 부검에 대한 태도에 미치는 영향 요인을 파악한다.

## 연구 방법

### 1. 연구 설계

본 연구는 간호대학생의 부검에 대한 인식과 태도를 파악하고 부검에 대한 태도에 미치는 영향을 알아보기 위한 서술적 조사 연구이다.

### 2. 연구 대상

광주광역시에 소재하는 남부대학교 간호학과 1∼4학년에 재학 중인 간호대학생을 대상으로 연구의 목적을 이해하고 연구 참여에 자발적으로 동의한 간호대학생으로 하였다. 본 연구에 동의하지 않은 간호대학생들은 연구대상자에서 제외하였다. 연구대상자 수는 G*power 프로그램 3.1.9.7를 이용하여 산출하였으며, Cohen [29]을 참고하여 효과크기 .15 [30], 유의수준 .05, 검정력 .95, 일반적 특성 및 연구변수 8개를 적용하여 다중회귀분석에 필요한 최소 표본 수는 160명이었다. 탈락율 20%를 고려하여 모집한 200명 중 응답 내용이 불충분하거나 주요 정보가 누락된 17부를 제외하고, 총 183부를 최종적으로 분석에 사용하였다.

### 3. 연구 도구

#### 1) 일반적 특성

대상자의 일반적 특성에는 인구학적 특성과 부검제도 관련 특성으로 구성되었다[11]. 인구학적 특성으로는 성별, 연령, 종교를 조사하였으며, 부검제도 관련 특성으로는 검시조사관 직업 인지유무, 법의학(법의간호학) 교육 희망 유무, 부검시설 지역설치에 대한 인식을 조사하였다.

#### 2) 부검에 대한 인식

부검에 대한 인식을 측정하기 위해 Yang 등[7]이 개발한 도구를 이메일을 통해 저자의 도구 사용 승인을 받고 일부 수정, 보완하여 사용하였다. 이 도구는 2018년 대한법의학회 변사 가이드라인을 기반으로 개발되었으며[9], 수정•보완된 도구는 간호학 및 법의학 전문가 3인에게 내용 타당도 검토를 받아 최종 확정되었다. 측정도구는 총 21문항으로 부검에 대한 인식 11문항, 부검에 대한 거부감 10문항으로 구성되었다. 각 문항은 Likert 5점 척도(매우 그렇다=5점, 조금 그렇다=4점, 그저 그렇다=3점, 별로 그렇지 않다=2점, 전혀 그렇지 않다=1점)으로 측정되었다. 점수가 높을수록 부검에 대한 긍정적인 인식과 경험을 나타낸다. Yang 등[7]의 연구에서 도구의 신뢰도는 Cronbachs′ α = .87이었으며, 본 연구에서는 Cronbachs′ α = .88로 나타났다.

#### 3) 부검에 대한 태도

부검에 대한 태도를 측정하기 위하여 Botega 등[24]이 개발하고, Oh [27]가 간호학생에 맞도록 번안하여 수정•보완한 도구를 이메일을 통해 저자의 승인을 얻어 사용하여 측정하였다. 이 도구는 총 17문항으로 부검에 대한 일반적 태도 11문항, 고인가족과 라포 2문항, 부검에 대한 정서적 반응 4문항으로 구성되었다. 각 문항은 Likert 5점 척도(완전 반대=5점, 거의 반대=4점, 중립=3점, 거의 동의=2점, 완전 동의=1점)으로 측정되었다. 부정문에 대한 동의를 역으로 환산하여 합산 점수가 높을수록 부검에 대한 일반적인 태도와 고인가족과 라포 및 정서반응이 긍정적임을 의미한다. Oh [27]의 연구에서 신뢰도는 Cronbach′s α = .71이었으며, 본 연구에서는 Cronbach′s α = .80으로 나타났다.

### 4. 자료 수집

본 연구자는 광주광역시 남부대학교의 기관생명윤리위원회(IRB) 승인을 받은 후 2024년 9월 2일부터 10월 4일까지 자료를 수집하였다. 간호학과 홈페이지 정보광장 자유게시판에 모집 공고문과 온라인 구글 링크 및 QR Code가 포함된 연구 설명서와 동의서를 업로드하여 대상자에게 연구의 목적과 연구 방법을 안내하였다. 참여자는 온라인 동의서를 작성한 후 설문에 참여할 수 있었으며, 동의하지 않을 경우 설문이 진행되지 않도록 설계하였다. 설문 응답은 언제든지 중단할 수 있도록 하였으며, 모든 응답을 완료하지 않고 제출 버튼을 누르지 않으면 해당 응답은 자동으로 저장되지 않도록 하였다. 본 연구에 총 200명의 간호대학생이 구글 설문을 통해 자발적으로 참여하였다. 그러나 일률적인 응답 등 불성실한 자료 17건을 제외하였고, 최종적으로 183명의 자료를 분석에 사용하였다.

### 5. 자료 분석

수집된 자료는 SPSS version 28.0(IBM Corp., Armonk, NY, USA)을 사용하여 유의수준 .05에서 양측 검정을 통해 분석하였다. 연구대상자의 일반적 특성과 검시조사관 직업 인지유무, 법의학(법의간호학) 교육 희망 유무, 부검시설 설치 여부는 빈도와 백분율, 평균과 표준편차를 이용하여 분석하였다. 연구대상자의 일반적 특성에 따른 부검에 대한 인식과 태도의 차이는 왜도(절대값>3), 첨도(절대값>10)로 정규성 검정[31] 후 Independent t-test와 one-way ANOVA로 검증하고 사후 검정은 Scheffé test로 분석하였다. 연구대상자의 부검에 대한 인식과 태도 간의 상관관계는 Pearson correlation coefficients를 이용하여 분석하였다. 연구대상자의 부검에 대한 태도에 미치는 영향 요인을 확인하기 위하여 다중회귀분석(multiple regression)을 이용하여 분석하였다. 단변량 분석에서 부검에 대한 태도에 유의한 관련성이 있는 것으로 나타난 연령, 법의학(법의간호학) 교육의 필요성, 부검시설 지역설치에 대한 인식, 부검에 대한 인식을 예측변수로 하여 다중회귀분석을 실시하였다. 예측변수 중 법의학(법의간호학) 교육 희망 유무, 부검시설 지역설치에 대한 인식, 부검에 대한 인식은 가변수(dummy variable) 처리하였고, 연령은 연속변수로 투입하였다.

### 6. 윤리적 고려

본 연구는 2024년 7월 광주광역시 남부대학교의 IRB 심의를 통과한 후 (IRB No. 1041478-2024-HR-001) 시행되었다. 윤리적 고려를 위하여 비밀보장을 약속하였으며, 연구 참여는 자발적인 동의에 기반하며, 연구 중단을 원할 시 언제든지 철회할 수 있음을 안내하였다. 연구 철회에 따른 어떠한 불이익도 없음도 명시하였다. 설문 작성에는 15∼20분이 소요되었으며, 참여완료 시 소정의 모바일 쿠폰을 사례로 제공하였다. 수집된 자료는 학술적인 목적으로만 사용되었으며, 연구자만 접속 가능한 비밀번호가 설정된 컴퓨터에 보관하였다. 대상자 고유번호를 부여하여 코딩 된 엑셀파일은 개인정보를 보호를 위해 비밀번호가 설정된 컴퓨터에서 보관하였다. 자료는 연구 종료 후 3년간 보관 후 복원 불가능한 방법으로 전자문서를 안전하게 파기할 것이다.

## 연구 결과

### 1. 일반적 특성

본 연구에 참여한 간호대학생(N=183) 중 142명(77.6%)이 여성이었으며, 평균연령은 21.23 ± 3.58세였다. 연령대별로는 19∼20세가 105명(57.4%), 21∼24세가 57명(31.1%), 25세 이상 21명(11.5%)이었다. 종교 여부에 대해서는 126명(68.9%)이 종교가 없다고 응답하였다. 검시조사관 직업에 대한 인식 여부는 ‘들어본 적 있다’고 응답한 대상자가 93명(50.8%)으로 가장 많았으며, 법의학(법의간호학) 교육 희망 유무에 대해서는 154명(84.2%)이 ‘필요하다’고 응답하였다. 또한, 부검시설 지역설치에 대한 인식은 ‘조건부 찬성한다’는 응답이 72명(39.5%)이었다(Table 1).

### 2. 대상자 특성에 따른 부검에 대한 인식과 태도

대상자의 부검에 대한 인식의 하위 영역별 점수로 부검에 대한 일반적 인식 점수는 평균 4.63 ± 0.43점이었고, 부검에 대한 거부감 정도는 평균 2.49 ± 0.79점이었다. 부검에 대한 태도의 하위 영역별 점수로 부검에 대한 일반적 태도가 평균 3.06 ± 0.51점, 고인 가족과 라포는 평균 3.27 ± 0.91점, 부검에 대한 정서적 반응 정도는 평균 3.35 ± 0.63점으로 나타났다(Table 2).

### 3. 일반적 특성에 따른 부검에 대한 인식과 태도의 차이

본 연구에 참여한 간호대학생의 일반적 특성에 따른 부검에 대한 태도를 분석한 결과, 연령(F = 3.75, *p* = .025), 법의학(법의간호학) 교육 희망 유무(t = 3.02, *p* = .003), 부검시설 지역설치에 대한 인식(F = 3.45, *p* = .018)에서 유의한 차이가 있었다. 연령에 따른 사후검정 결과, 19~20세인 경우보다, 25세 이상인 그룹에서 부검에 대한 태도 점수가 유의미하게 높은 것으로 나타났다. 법의학(법의간호학) 교육 희망 유무는 ‘필요하다’고 응답한 경우, 그리고 부검시설 지역설치에 대한 인식에 ‘찬성한다’의 경우 부검에 대한 태도 점수가 높게 나타났다(Table 3).

### 4. 부검에 대한 인식과 태도 간의 상관관계

대상자의 부검에 대한 인식, 부검에 대한 거부감, 부검에 대한 일반적 태도, 고인 가족과 라포, 부검에 대한 정서반응 간의 상관성을 파악하기 위해 Pearson 상관분석을 실시하였다. 분석 결과, 부검에 대한 일반적 태도(r = .18, *p* < .001), 고인 가족과 라포(r = .17, *p* < .001)는 부검에 대한 인식과 유의한 양의 상관관계를 보였다. 반면, 부검에 대한 거부감(r = −.18, *p* < .001)은 부검에 대한 인식과 유의한 음의 상관관계를 나타냈다. 부검에 대한 거부감은 고인 가족과 라포(r = −.10, *p* < .001)과 유의한 음의 상관관계를 나타났고, 고인 가족과 라포는 부검에 대한 정서반응(r = .24, *p* < .010)과 유의한 양의 상관관계를 보였다(Table 4).

### 5. 부검에 대한 태도에 미치는 영향

회귀분석을 수행하기 전, 오차의 자기상관과 다중공선성을 검토한 결과, Durbin-Watson을 이용한 오차의 자기상관은 2.05로 검정통계량 2 이상의 값을 보여 자기상관이 없이 독립적인 것으로 확인되었다. 변수들 간의 다중공선성 여부를 검정한 결과, 분산팽창지수는 1.06∼1.15로 10.0을 초과하지 않았으며, 공차한계(tolerance)는 0.87∼0.95로서 0.1 이상의 값을 보여 다중공선성의 문제가 없음을 확인하였다. 또한 정규성과 선형관계의 가정을 만족하였고, 특이값을 검토하기 위한 Cook’s 거리 값은 모든 개체에서 1.0을 초과하지 않아 분석 가정을 충족하였다.

부검에 대한 태도에 미치는 영향 요인을 파악하기 위하여 다중회귀분석을 실시한 결과, 유의한 영향요인은 부검에 대한 인식 (β = .29, *p* < .001), 부검시설 지역설치에 대한 인식 (β = .16, *p* = .025), 법의학(법의간호학) 교육 희망 유무 (β = .16, *p* = .028)순으로 나타났다. 연령 (β = .12, *p* = .098), 및 기타 변수는 유의미한 영향을 미치지 않았다. 본 회귀분석 모형은 통계적으로 유의하였으며 (F = 9.10, *p* < .001), 주요 변수의 설명력은 17%로 나타났다(Table 5).

## 논의

본 연구는 간호대학생의 부검에 대한 인식과 태도를 확인하고, 간호대학생의 부검에 대한 태도에 영향을 미치는 요인을 분석하며, 간호대학생의 긍정적인 부검 태도 향상을 위한 프로그램 개발의 근거 자료로 활용되기 위해 시행하였다. 기존의 부검 연구는 대부분 법의학을 기초로 한 의과대학생들을 대상으로 진행되었지만, 본 연구는 간호대학생에게 적용하여 기초간호교육 강화의 일환으로 법의학 기반의 법의간호학의 필요성을 강조할 수 있을 것이다[13].

연구대상자의 일반적 특성에 따른 부검에 대한 태도는 연령, 법의학(법의간호학) 교육 희망 유무, 부검시설 지역설치에 대한 인식이 통계적으로 유의한 차이를 보였다. 25세 이상 연령층에서 부검에 대한 태도가 유의미하게 긍정적이었다. 이는 간호대학생의 전공 변경 및 편입 증가로 연령대가 다양화되고 있으나[32], 부검 태도와 관련된 선행 연구가 부족해 결과를 비교하기는 어려운 상황이다. 한편, 일반인을 대상으로 한 연구에서는 연령이 높은 집단일수록 부검시설의 지역 설치에 대한 인식이 낮은 것으로 확인되었다[11].

법의학(법의간호학) 교육 희망 유무에 따른 부검에 대한 태도는 ‘필요하다’ 고 느낄수록 긍정적이었으며, 이는 일반 대중에게 법의학(법의간호학)에 대한 흥미를 유발하는 영상매체들이 주로 허구적인 상황을 기반으로 제작되므로 그릇된 인식을 심어줄 위험이 있음을 시사한다. 따라서 법의학(법의간호학)에 정확한 지식전달과 체계적인 교육이 필요하다는 점을 강조할 수 있다[13]. 이러한 결과는 의과대학생을 대상으로 한 연구에서도 일치하는 결과로[2,7,25,26], 법의학 관련 교육을 통해 부검에 대한 지식과 이해가 확장되고 거부감이 줄어드는 긍정적인 변화가 나타났기 때문으로 해석된다.

부검시설 지역 설치에 대한 인식에서 반대하는 응답자가 찬성하는 사람들에 비해 부검에 대한 태도가 유의미하게 낮은 점수를 보였다. 이는 간호대학생을 대상으로 한 관련 연구는 아직 없지만, 일반인을 대상으로 한 연구에서는 부검시설이 본인 거주 지역에 들어오는 것을 어떠한 경우에도 반대한다는 응답이 38%에 달하며[11], 부검시설에 대한 부정적인 인식이 여전히 존재함을 보여준다[11]. 그러므로 각 검시주체들과 정부기관은 이러한 문화적 인식 변화를 위한 정책적 논의가 필요하다고 본다.

부검에 대한 태도는 부검에 대한 인식과 유의한 상관관계를 보였으며, 선행 연구에서도 법의학(법의간호학) 교육의 필요성에 대한 인식도가 상승하고 태도가 더 긍정적으로 변화되었다고 보고되었다[15,21]. 이러한 점에서 간호사들 또한 법의학(법의간호학)교육과 부검의 필요성을 느끼고 있다고 사료된다[14,21]. 즉 간호대학교에서 체계적인 법의학(법의간호학)교육을 시행한다면, 간호사의 부검에 대한 개념이 명확히 정립되어 유가족의 부검 결정 과정에 긍정적인 역할을 미칠 수 있다[16,20]. 이는 부검의 활성화와 유가족 지원 측면에서 중요한 역할을 할 수 있을 것이다.

부검에 대한 태도에 영향을 미치는 주요 요인은 ‘부검에 대한 인식’으로 나타났으며, 부검에 대한 태도가 부검에 대한 인식으로 설명되는 정도는 17%로 낮게 나타났다. 이는 몇 가지 요인에 기인할 수 있다. 첫째, 측정 도구가 태도와 인식을 포괄적으로 측정하지 못했거나, 정서적 요인, 윤리적 가치관, 종교적 배경 등 중요한 변수를 포함하지 않았을 가능성이 있다. 둘째, 본 연구가 특정 대학교 간호대학생만을 대상으로 하여 표본의 다양성이 부족했던 점이 설명력에 영향을 미쳤을 수 있다. 셋째, 부검에 대한 태도는 단순히 인식으로만 형성되는 것이 아니라 교육 경험, 사회적 규범, 주변 환경 등 다양한 요인의 영향을 받는 복합적인 과정일 가능성이 있다. 이러한 한계는 후속 연구를 통해 보완할 필요가 있다.

특히, 간호학생을 대상으로 한 연구에서 부검에 대한 태도의 하위영역인 정서적 반응을 긍정적인 태도로 본 연구와[23]. 법의학 관련 교육 경험이 있는 간호대학생의 부검 필요성 인식을 본 연구결과가 있었지만[13], 간호대학생의 부검에 대한 태도에 영향을 미치는 요인을 규명한 연구는 부족한 실정이다. 다만, 일반대학생과 의과대학생의 부검에 대한 인식도 조사에서 일반대학생이 부검에 대해 긍정적인 태도를 보였다는 연구[22]와 대학병원 간호사가 부검에 대한 지식과 경험이 높음에도 불구하고 필요성 인식은 낮았다는 연구[20]와 맥락을 같이 한다.

간호사가 유가족에게 부검의 필요성을 설명하고 동의를 얻는 과정에서 중요한 역할을 한다는 점과[13,14], 검시조사관으로서 변사자 검시, 의료사고 의심 사례 조사, 유가족 면담 등을 수행하기 위해 의학적·법적 관점에서 긍정적인 부검 인식을 갖추는 것은 필수적이다[10]. 이러한 배경에서 간호사들은 다양한 환경에서 사망사건 발생 시 유가족을 지원하고 부검을 촉진하기 위해 올바른 태도가 강조된다[21,24,28].

따라서, 간호대학생들에게 법의학(법의간호학) 교육을 제공함으로써 유가족의 부검 결정 과정에서 간호사가 신뢰받는 조력자로서 역할을 효과적으로 수행할 수 있을 것이다[23]. 이는 간호사가 단순히 법의학적 지식을 확장하는 것을 넘어, 감정적 지지를 통해 유가족과 공감하며 부검에 대한 긍정적 태도를 이끌어내는 데 기여할 것이다[14].

선행 연구 결과, 국가별 사망 원인의 정확한 확인을 위해 검시 건수 대비 부검 비율을 비교한 결과, 전담 검시 제도를 운영하는 영국에서는 전체 검시 중 47.1%에 대해 부검이 이루어지는 반면, 우리나라에서는 12.6%에 불과하다는 점이 드러났다. 이러한 차이는 제도의 구조적 차이에서 비롯된 것으로 해석된다. 영국의 전담 검시 제도는 법적으로 검시 대상을 명확히 규정하고, 검시 전문가를 배치하여 철저하게 사망 원인을 확인하는 데 중점을 둔다. 반면, 우리나라는 범죄와 관련된 변사체를 중심으로 사망 원인을 규명하는 사법 검시 제도를 운영하고 있어 검시율과 부검률이 상대적으로 낮은 결과를 보이고 있다[6].

한국에서는 장례 문화의 특성상 부검이 일반적으로 3일장 내에 신속히 이루어진다. 이는 유족들의 요청으로 인해 부검 절차가 서둘러지는 경우가 많아 수사기관에 시간적 압박을 가중시키며, 정밀하고 철저한 부검이 어려워지는 요인으로 작용한다[11]. 일본에서도 변사체로 간주되는 시간이 짧고, 장례 문화상 2~3일 후 화장을 진행하는 경우가 많아 범죄성이 강하게 의심되는 사건에만 사법해부가 이루어져 진실 규명에 어려움이 있다. 독일은 부검율이 감소하는 추세인데, 이는 공공 안전에 대한 인식 부족과 정치적 요인, 그리고 연구•교육 지원의 부족 등 재정적 문제가 주요 원인으로 지목된다[6,11].

부검에 대한 태도와 인식의 전환은 단기간에 이루어질 수 없는 문제로, 문화적 배경과 사회적 인식을 고려한 장기적인 노력이 필요하다. 이를 위해 정책적 접근이 논의될 필요가 있다[6,11,28].

본 연구를 통해 간호대학생의 부검에 대한 인식이 부검에 대한 태도에 영향을 미치는 주요 요소임을 확인하였다. 따라서 향후 연구에서는 전국 간호대학생의 다양한 특성을 반영하여 부검에 대한 태도와 관련하여 다양한 영향요인을 규명하는 연구가 지속적으로 확대되어야 할 것이다. 또한 간호대학생의 부검에 대한 긍정적인 태도를 향상하기 위하여 영향요인을 반영한 교육프로그램이 운영될 수 있도록 행정적, 교육적 지원이 뒷받침되어야 할 것이다.

## 결론

본 연구는 광주광역시 소재 남부대학교 간호학과에 재학 중인 간호대학생 183명을 대상으로 부검에 대한 인식과 태도를 파악하고, 부검에 대한 태도에 미치는 영향을 알아보고자 시도되었다. 연구 결과, 부검에 대한 인식이 태도에 영향을 미치며 설명력은 17%로 나타났다. 이를 바탕으로 간호대학생의 부검에 대한 긍정적인 태도를 형성하기 위해 체계적인 법의학 교육 및 인식 개선 프로그램 개발이 필요함을 확인하였다. 연구에 제한점인 표본의 지역성과 모집방식의 제약을 넘어 연구 결과의 일반화를 위해 광범위한 대상과 반복 연구를 제언한다. 또한, 부검 태도 측정을 위한 표준화된 부검에 대한 태도 측정 도구의 개발과 학년별 눈높이에 맞는 프로그램 적용이 필요하다. 본 연구는 기초간호교육과 실무에 자료를 제공함으로써 전문직 간호 발전에 기여할 것으로 기대된다.

## Figures and Tables

**Table 1.** Participant’s Characteristics (N = 183)

Variables	Categories	n	(%)	M ± SD
Sex	Men	41	(22.4)	
	Women	142	(77.6)	
Age (yr)	19~20	105	(57.4)	21.23 ± 3.58
	21~24	57	(31.1)	
	≥ 25	21	(11.5)	
Religion	None	126	(68.9)	
	Protestantism	44	(24.0)	
	Catholicism	6	(3.3)	
	Buddhism	7	(3.8)	
MDI recognition	Yes	55	(30.1)	
	No	35	(19.1)	
	Unknown	93	(50.8)	
Necessity of forensic medicine (forensic nursing)	Yes	154	(84.2)	
	No	29	(15.8)	
Installation of autopsy facilities	Agree	39	(21.4)	
	Agree conditionally	72	(39.5)	
	Disagree	12	(6.2)	
	Irrelevance	60	(32.9)	

M = Mean; SD = Standard deviation; MDI = Medicolegal death investigation.

**Table 2.** Perceptions of and Attitudes toward Autopsies (N = 183)

Variables	Categories	Number of items	Min	Max	M ± SD	Range
Perceptions of autopsies	General perceptions of autopsies	11	1.00	5.00	4.63 ± 0.43	1.00~5.00
	Opposing autopsies	10	1.00	5.00	2.49 ± 0.79	1.00~5.00
Attitudes toward autopsies	General attitudes toward autopsies	11	1.00	5.00	3.06 ± 0.51	1.00~5.00
	Rapport with the patient’s family	2	1.00	5.00	3.27 ± 0.91	1.00~5.00
	Emotional responses toward autopsies	4	1.00	5.00	3.35 ± 0.63	1.00~5.00

Min = Minimum; Max = Maximum; M = Mean; SD = Standard deviation.

**Table 3.** Differences in Perceptions of Autopsies and Attitudes toward Autopsies according to General Characteristics (N = 183)

Variables	Categories	Perceptions of autopsies		Attitudes toward autopsies	
		M ± SD	t/F(*p*)	M ± SD	t/F(*p*)
			Scheffé		Scheffé
Sex	Men	75.07 ± 9.85	−0.71 (.481)	54.39 ± 9.17	0.73 (.467)
	Women	76.13 ± 8.04		53.34 ± 7.84	
Age (yr)	19~20^c^	76.33 ± 8.31	0.84 (.433)	52.21 ± 7.38	3.75 (.025) a > c
	21~24^b^	74.72 ± 8.85		55.07 ± 9.28	
	≥ 25^a^	76.90 ± 8.17		56.33 ± 7.38	
Religion	None	76.20 ± 8.50	1.77 (.154)	53.75 ± 8.36	.12 (.951)
	Protestantism	76.48 ± 7.57		53.36 ± 8.30	
	Catholicism	69.17 ± 4.79		52.00 ± 4.52	
	Buddhism	72.57 ± 13.25		53.00 ± 6.40	
MDI recognition	Yes	74.91 ± 8.64	0.61 (.542)	53.37 ± 7.61	2.22 (.112)
	No	75.83 ± 8.98		55.44 ± 8.49	
	Unknown	76.51 ± 8.18		52.55 ± 8.01	
Necessity of forensic medicine (forensic nursing)	Yes	75.74 ± 8.20	−0.57 (.567)	54.34 ± 8.08	3.02 (.003)
	No	76.72 ± 9.81		49.48 ± 7.30	
Awareness of local installation of autopsy facilities	Agree^a^	75.59 ± 9.29	0.62 (.601)	56.92 ± 9.62	3.45 (.018) a > d
	Agree conditionally^b^	75.85 ± 77.71		53.51 ± 7.76	
	Disagree^c^	79.08 ± 10.75		51.67 ± 10.16	
	Irrelevance^d^	75.52 ± 8.35		51.85 ± 6.48	

M = Mean; SD = Standard deviation; MDI = Medicolegal death investigation.

**Table 4.** Correlations among Perceptions of and Attitudes toward Autopsies (N = 183)

Variables	Perceptions of autopsies		Attitudes toward autopsies		
	General perceptions of autopsies	Opposing autopsies	General attitudes toward autopsies	Rapport with the patient’s family	Emotional reaction to the necropsy
	r (*p*)				
Opposing autopsies	−.18 (< .001)	1	-	-	-
General attitudes toward autopsies	.18 (< .001)	−.43	1	-	-
Rapport with the patient’s family	.17 (< .001)	−.10 (< .001)	.34	1	-
Emotional reaction to the necropsy	.07	−.29	.59	.24 (< .010)	1

**Table 5.** Factors Influencing Nursing Students' General Attitudes toward Autopsies (N = 183)

Variables	B	SE	β	t	*p*
(Constant)	51.59	3.82		13.49	< .001
Age (yr)	0.92	.56	.12	1.66	.098
Necessity of forensic medicine (forensic nursing)	2.36	1.06	.16	2.22	.028
Awareness of local installation of autopsy facilities	1.02	.45	.16	2.27	.025
Awareness of autopsies	0.19	.15	.29	4.19	< .001
R	.41				
R^2^ (Adj R^2^)	.17 (.15)				
F (*p*)	9.10 (< .001)				

SE = Standard error.

## Data Availability

Please contact the corresponding author for data availability.
